# Shared glycosylation-related molecular mechanism and immune infiltration patterns of fetal growth restriction and preeclampsia

**DOI:** 10.3389/fimmu.2026.1791113

**Published:** 2026-07-23

**Authors:** Tingxuan Yin, Xianyang Hu, Hailin Yu, Tianqi Zhang, Qin Yu, Xixi Huang, Chunfang Xu, Chuanling Tang, Lu Liu, Meirong Du

**Affiliations:** 1 Shanghai Key Lab of Reproduction and Development, Shanghai Key Lab of Female Reproductive Endocrine Related Diseases, Obstetrics & Gynecology Hospital of Fudan University, Shanghai, China; 2 Center for Assisted Reproduction, Shanghai Key Laboratory of Maternal Fetal Medicine, Shanghai Institute of Maternal-Fetal Medicine and Gynecologic Oncology, Shanghai First Maternity and Infant Hospital, School of Medicine, Tongji University, Shanghai, China; 3 Shanghai Key Laboratory of Maternal Fetal Medicine, Shanghai Institute of Maternal-Fetal Medicine and Gynecologic Oncology, Shanghai First Maternity and Infant Hospital, School of Medicine, Tongji University, Shanghai, China; 4 State Key Laboratory of Quality Research in Chinese Medicine and School of Pharmacy, Macau University of Science and Technology, Macao, Macao SAR, China

**Keywords:** fetal growth restriction, preeclampsia, glycosyltransferase, machine learning, immune infiltration, WGCNA, placenta, nomogram

## Abstract

**Background:**

Placental dysfunction and immune dysregulation are central to disorders such as fetal growth restriction (FGR) and preeclampsia (PE). Although aberrant placental glycosylation has been implicated in various pregnancy complications, the specific expression patterns of glycosylation-related genes (GRGs) associated with placental pathology and their potential role in driving immune imbalance at the maternal−fetal interface remain poorly understood.

**Methods:**

First, differentially expressed gene (DEG) identification and gene set enrichment analysis based on two GEO datasets (GSE114691 and GSE203507) revealed disease-associated pathways. Weighted gene co-expression network analysis (WGCNA) further identified glycosylation-related gene modules significantly associated with disease status. Three machine learning algorithms (LASSO, random forest, and support vector machine) were employed to screen diagnostic key genes, and a predictive nomogram was constructed. The efficacy of the diagnostic model was evaluated using receiver operating characteristic curves, calibration curves, and decision curve analysis. CIBERSORT was used to assess immune cell infiltration characteristics in the placenta and decidua of FGR, PE, and PE+FGR. Furthermore, single-cell RNA sequencing validated the expression patterns of key diagnostic genes across different trophoblast subsets, and cell-cell communication networks were analyzed using CellChat. In addition, this study validated the diagnostic efficacy of key genes in real-world samples.

**Result:**

Following batch correction and integration of two datasets, 104 samples were analyzed, including controls (n=26), PE (n=28), FGR (n=23), and PE+FGR (n=27). Glycosylation-related pathways were enriched in disease groups. Intersection of WGCNA modules with glycosylation genes yielded 97 overlapping genes, from which five core genes were identified using three machine learning algorithms. A diagnostic model based on these genes showed good predictive performance across disease groups. Immune infiltration analysis implicated multiple immune cell types in disease pathogenesis. Real-world validation of expression of B3GNT2 and ST6GAL1 differed significantly between normal and PE/FGR groups.

**Conclusion:**

Five candidate hub genes (B3GNT2, ST6GAL1, GALNT11, GCNT4, and LARGE2) were identified and a nomogram was constructed for FGR, PE, and PE+FGR diagnosis. Protein-level validation confirmed significant downregulation of B3GNT2 and ST6GAL1 in placental tissues from FGR and PE patients; mRNA-level validation of GALNT11, GCNT4, and LARGE2 is provided in independent clinical samples. These hub genes were associated with key immune cell types at both the placenta and decidua.

## 1 Introduction

Fetal growth restriction (FGR) and preeclampsia (PE) are two predominant pregnancy complications that pose formidable threats to maternal and fetal health worldwide. Clinically, PE is characterized by new-onset hypertension accompanied by proteinuria after 20 weeks of gestation, whereas FGR is defined by the failure of the fetus to reach its genetically determined growth potential (1). Notably, their co-occurrence (PE+FGR) represents the most severe manifestation within the spectrum of placental disorders (2). Patients with the PE+FGR phenotype face significantly higher risks of perinatal mortality and adverse long-term cardiovascular and metabolic outcomes.

The shared pathophysiological foundation of these disorders is widely attributed to impaired trophoblast invasion and inadequate remodeling of the uterine spiral arteries. This failure in vascular adaptation, often rooted in early decidualization defects and hormonal dysregulation during the secretory phase and early pregnancy, lead to chronic placental ischemia and a systemic antiangiogenic state (3). This state is characterized by elevated soluble fms-like tyrosine kinase-1 (sFlt-1) and soluble endoglin (sEng), alongside decreased placental growth factor (PlGF) (4). Despite these shared pathways, the precise molecular drivers that differentiate isolated FGR or PE from the more catastrophic PE+FGR phenotype, and how these drivers bridge intrinsic placental dysfunction with dysregulated maternal immune responses, remain poorly understood.

The survival of the semi-allogeneic embryo and the establishment of immune tolerance are critical for maintaining a normal pregnancy and rely on effective maternal-fetal communication. Glycosylation, one of the most prevalent and functionally significant post-translational modifications, plays a pivotal role in mediating this dialogue (5). Terminal glycan epitopes, including fucosylated and sialylated oligosaccharide structures, govern cell-cell and cell-extracellular matrix interactions critical for implantation and placental development. Components of the maternal-fetal interface, including the endometrial epithelium, decidual stroma, and trophoblasts, are enriched in glycoconjugates and glycan-binding molecules that are essential for successful pregnancy (5). Abnormal glycosylation is associated with various pregnancy complications. Changes at the placental O/N-glycome caused by disrupted activities of glycosidases and glycosyltransferases in endoplasmic reticulum and Golgi apparatus, or steric interference at glycoproteins, have been associated with pathologies that include FGR, PE and miscarriage (5–8). However, most glycobiology studies in reproductive process remain at the level of glycan profile association analysis (7). The mechanistic links between aberrant glycosylation and pathological gestations, particularly the specific roles of glycosyltransferases in placental dysfunction, remain insufficiently explored.

In this study, we employed an integrated approach combining multi-omics data with artificial intelligence algorithms to systematically map the glycosylation-related genes (GRGs) regulatory landscape in PE, FGR, and PE+FGR. By applying Weighted Gene Co-expression Network Analysis (WGCNA) and multiple machine-learning methods, including least absolute shrinkage and selection operator (LASSO), support vector machine-recursive feature elimination (SVM-RFE), and random forest, to transcriptomic data from placental tissue samples, with paired decidua basalis data used for immune infiltration correlation analysis, we identified key diagnostic biomarkers specific to disease severity. These findings were further validated using single-cell RNA sequencing (scRNA-seq) to determine cellular specificity. CIBERSORT analysis was employed to explore how GRG dysregulation reshapes the immune microenvironment. Finally, the core biomarkers were experimentally validated in independent clinical cohorts via quantitative real-time PCR (qPCR), immunohistochemistry (IHC) and western blot (WB).

We hypothesize that dysregulation of GRGs, particularly those within the sialylation pathway, disrupts trophoblast-immune crosstalk at the maternal-fetal interface, potentially serving as a common molecular link between placental defects and maternal immune maladaptation.

## 2 Methods

### 2.1 Mendelian randomization analysis

Bidirectional two-sample Mendelian randomization (MR) was performed to assess the causal relationship between PE and FGR, thereby motivating the integrated analysis of the two conditions. Summary statistics were obtained from large-scale genome-wide association studies (GWAS) for PE and FGR. Genetic instruments for PE were derived from a GWAS including 267,242 European individuals (2,355 PE cases; ebi-a-GCST90018906, PMID: 34594039). FGR data were obtained from the FinnGen R12 dataset (finngen_R12_P16_SLOW_FETAL_GROWTH_FETAL_MALNUTRITION), which defines FGR based on ICD-10 codes for fetal growth retardation and malnutrition. Single nucleotide polymorphisms associated with exposures at P < 1×10^-5^ were selected as instrumental variables. Bidirectional two-sample Mendelian randomization (MR) was conducted using the inverse variance weighted (IVW) method as the primary analysis.

### 2.2 Data processing and identification of differentially expressed genes

**Figure 1:**
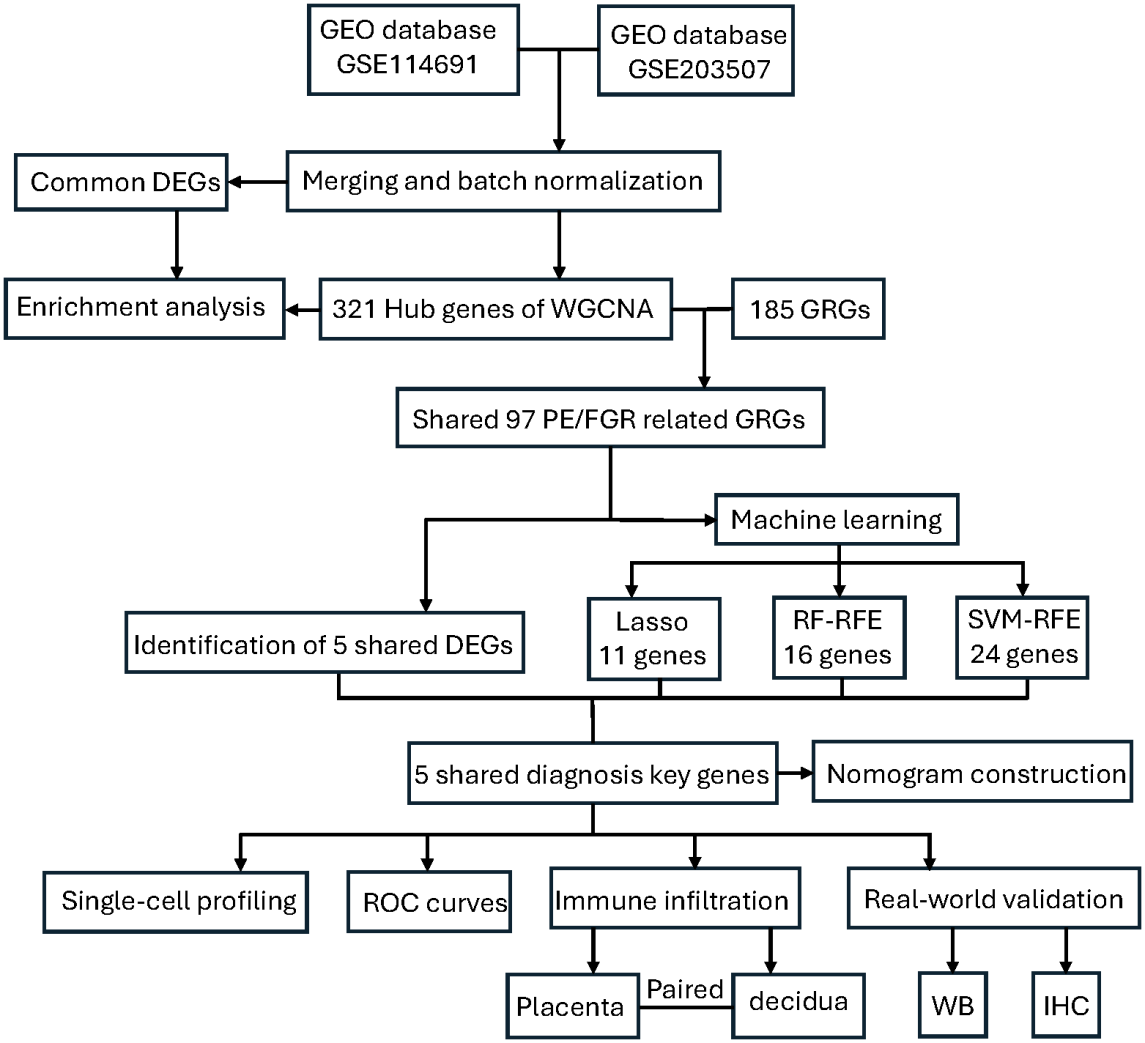
The flow chart for the whole design. DEGs, differentially expressed genes. WGCNA, weighted gene co-expression analysis. RF-RFE, random forest-recursive feature elimination. SVM-RFE, support vector machine-recursive feature elimination; LASSO, least absolute shrinkage and selection operator; IHC, immunohistochemical.

Gene expression microarray datasets GSE114691 (9) and GSE203507 (10) were obtained from the Gene Expression Omnibus (GEO) database for placental tissue analysis. The GSE114691 dataset, based on the GPL11154 platform, comprises placental tissue samples from four clinical groups: FGR (n=20), PE (n=20), PE+FGR (n=18), and normal pregnancy (control, n=21). The GSE203507 dataset, based on the GPL16791 platform, includes paired decidual and placental samples from four groups: Early-Onset PE (EOPE, n=8), PE+FGR (n=7), FGR (FGR, n=5) and uncomplicated term births (TB, n=5). Samples from the two datasets were integrated for downstream analysis. By applying R packages “limma” and “sva”, we standardized the data in datasets, performed batch effect correction, and screened differentially expressed genes (DEGs). The cut-off criteria were adjusted P < 0.05 and |log2 fold change (FC)| > 0.5. This relaxed fold-change threshold was intentionally chosen for the integrated multi-dataset analysis to capture consistently dysregulated genes across heterogenous platforms while maximizing sensitivity for shared disease signatures. The heatmap and volcano diagram were obtained using the “ggplots” R package. The data analysis workflow is shown in **Figure 1**. And the information on microarray datasets obtained from GEO was listed in **Supplementary Table 1**.

### 2.3 Enrichment analysis

Gene Ontology (GO) and Kyoto Encyclopedia of Genes and Genomes (KEGG) pathway enrichment analyses were performed using the “clusterProfiler” and “pathview” R packages to predict the potential function of DEGs between two groups. The potential underlying molecular mechanisms of DEGs were further investigated by applying gene set enrichment analysis (GSEA). In addition, we performed disease ontology (DO) enrichment analysis of DEGs using the “Dose” R package. Gene-concept networks and pathway-gene interaction networks were constructed and visualized using the ‘enrichplot’ and ‘igraph’ R packages to elucidate the complex relationships between key genes and enriched functional terms.

### 2.4 Weighted gene co-expression network analysis

Using the ‘WGCNA’ R package, potential GRG modules associated with PE and FGR were identified. The data were preprocessed by filtering out genes with low variance, retaining only those with variance greater than the median. Missing values in the dataset were checked, and genes or samples with excessive missing data were removed to ensure the integrity of the analysis. Hierarchical clustering was performed on the samples to detect potential outliers, with samples outside a predefined cutoff being excluded. Gene modules were identified using hierarchical clustering based on the Topological Overlap Matrix (TOM). The optimal soft-thresholding power was determined, with a scale-free topology criterion set at R² > 0.85. Given the complexity of the integrated multi-platform dataset (n=104), a soft-thresholding power of β = 18 was required to meet this criterion, which is consistent with previously reported values for large integrated transcriptomic datasets. The TOM was then calculated, followed by hierarchical clustering of genes to construct a dendrogram, which was used for module detection. Modules were identified using the dynamic tree cutting method, with a minimum module size of 50 genes. The identified modules were correlated with clinical traits, such as survival data, using Pearson’s correlation. Significant modules were identified based on the correlation between gene module membership (MM) and gene trait significance (GS).

### 2.5 Hub gene identification and construction and evaluation of diagnostic model

In this study, hub genes were defined as overlapping genes from three machine learning algorithms, including LASSO using the “glmnet” R package, SVM-RFE using the “e1071” R package, and random forest using the “randomForest” R package. Based on the selected hub genes, we constructed a nomogram model through the “rms” package to predict the incidence of different diseases. The calibration curve and receiver operating characteristic (ROC) curve were drawn to evaluate the suitability of our nomogram for clinical use.

### 2.6 Gene set enrichment analysis and immunological correlation analysis

The CIBERSORT algorithm was applied using the LM22 reference matrix to estimate the relative proportions of 22 immune cell types across disease and control groups in placental tissue. Differences in immune cell infiltration among groups were visualized using box plots. LASSO regression analysis was then performed to identify key immune cell types associated with disease status. The correlation between key gene expression levels and immune cell infiltration was analyzed and visualized as a heatmap. For decidual immune infiltration analysis, the decidua basalis (DB) fraction of the GSE203507 dataset was used. GSE203507 contains paired villous tissue (VT) and decidua basalis (DB) RNA-seq data from the same patients across four clinical groups (EOPE n=8, EOPE+FGR n=7, preterm FGR n=5, TB n=5). CIBERSORT was applied separately to the DB expression matrix to estimate immune cell proportions in the decidua, and correlation analysis was then performed between placental hub gene expression levels and decidual immune cell fractions. It should be noted that the LM22 reference matrix is leukocyte-centric and does not include placenta-specific cell types such as trophoblasts; the estimated fractions therefore reflect relative immune composition rather than absolute cell abundance, which is consistent with its application in other tissue-based transcriptomic studies.

### 2.7 Single-cell data processing and cell communication analysis

In the downloaded datasets GSE282038 (11) and GSE173193 (12), cells with detected gene numbers between 500 and 3000 and mitochondrial UMIs less than 10% were retained. GSE282038 provides scRNA-seq data from EOPE+FGR placental trophoblasts; GSE173193 provides single-cell profiling of human placenta from PE and normal control groups. Genes expressed in fewer than three cells were excluded. Downstream data processing and analysis, including filtering, normalization, scaling, batch removal, and dimension reduction were performed using the Seurat package. We clustered all the cells based on the integrated gene expression matrix using Seurat with a parameter “Resolution = 0.5” and generated 18 clusters. To minimize different batches from healthy controls and disease groups, we used Harmony to verify the integration reliability. Cell type-specific genes were identified using the Wilcoxon rank-sum test within the FindAllMarkers function in Seurat on a log-transformed expression matrix. We identified DEGs between normal individuals and the patients with the Seurat “FindMarkers” command with the parameters “test.use” as Wilcox, “logfc.threshold” as 0.25 and adjusted p value as 0.05. The systematic analysis of cell communication was based on the network analysis and pattern recognition approaches provided by CellChat (version 0.0.1) R package.

### 2.8 Experimental validation in independent clinical cohorts

To validate the expression changes of the candidate hub genes, placental tissues were collected from August 2025 to December 2025 at the Obstetrics and Gynecology Hospital of Fudan University and Shanghai First Maternity and Infant Hospital, China. This study was approved by the Ethics Committees of the participating institutions (Ethics approval No. 2020-S218).

Placental tissues were collected from patients with FGR (n = 14), PE (n = 14), and uncomplicated controls (NP, n = 12). FGR was defined as a birthweight below the 10th percentile for gestational age. Preeclampsia was diagnosed according to the standard criteria: new-onset hypertension (blood pressure ≥ 140/90 mm Hg) accompanied by proteinuria (≥ 0.3 g/24 hours) after 20 weeks of gestation. Patients with preexisting major medical conditions (including diabetes and chronic hypertension), and pregnancies complicated by major fetal structural or chromosomal anomalies, were excluded. All placental tissues were collected during cesarean sections or spontaneous vaginal deliveries. Detailed clinical characteristics of all groups are listed in **Supplementary Table 2**.

Placental tissue samples (~1 cm³) were dissected from multiple regions on the maternal side near the umbilical cord insertion site and rinsed with sterile saline to remove residual maternal blood. Portions of the tissues were fixed in a 4% paraformaldehyde solution, embedded in paraffin, and subjected to IHC staining. The remaining samples were snap-frozen in liquid nitrogen and stored at −80 °C for WB and RT-qPCR analysis.

#### 2.8.1 Western blot

Placental samples were lysed with RIPA lysis buffer (Solarbio, China), and protein concentrations were quantified using a BCA protein quantification kit (NCM Biotech, China). Equal amounts of protein were separated on 10% SDS-PAGE gels and transferred onto PVDF membranes (Millipore, USA). After blocking with 5% BSA (Solarbio, China) for 1 hour, the membranes were washed three times with Tris-buffered saline containing 0.1% Tween-20 (TBST) and incubated overnight at 4 °C with primary antibodies against GAPDH (Sigma, USA; 1:10,000), B3GNT2 (Proteintech, China; 1:5,000), and ST6GAL1 (Proteintech, China; 1:5,000). Membranes were then incubated with HRP-conjugated secondary antibodies for 1 hour at room temperature, and protein bands were visualized using a chemiluminescence detection system (Thermo Fisher Scientific, USA). Band intensities were quantified by densitometry using ImageJ and normalized to GAPDH.

#### 2.8.2 RT-qPCR

Total RNA was extracted from frozen placental tissue using an EZB tissue RNA extraction kit (EZBioscience, USA) according to the manufacturer’s instructions. RNA concentration and purity were assessed using a NanoDrop spectrophotometer (Thermo Fisher Scientific, USA), and 500 ng of total RNA was reverse-transcribed into cDNA using Evo M-MLV RT Premix for qPCR (Accurate Biology, China). Quantitative real-time PCR was performed on a QuantStudio 12K Flex Real-Time PCR System (Applied Biosystems, USA) using Hieff^®^ qPCR SYBR Green Master Mix (Low Rox) (Yeasen, China) with the following cycling conditions: 95 °C for 5 min, followed by 40 cycles of 95 °C for 10 s and 60 °C for 30 s. GAPDH served as the internal reference gene. Relative mRNA expression was calculated using the 2^−ΔΔCt method, and all reactions were performed in triplicate. The primer sequences (5′→3′) used were: B3GNT2, forward ACTGGAACCGAGAGCAAGAGAA, reverse GTTAAAACCCGTAACCACCGAC; ST6GAL1, forward GGCAGGTGTGCTGTTGTGTCGT, reverse TTGTTGGAAGTTGGCTGTGGGT; GALNT11, forward GTTTTCTGCCTTGCTTCGGACA, reverse TCAACCCCTCACGCTTTGTATT; GCNT4, forward ACCTAAAGAGGAGCGAAGGGAT, reverse GGCTTAATGTTCTGGTGTGTGA; LARGE2, forward ATCCCCAACAAGCACTACTC, reverse TCACTCTGGTTCTCCACAAG; GAPDH, forward GTCTCCTCTGACTTCAACAGCG, reverse ACCACCCTGTTGCTGTAGCCAA.

### 2.9 Statistics analysis

All statistical analyses were conducted using R packages [R software (version 4.2.0)]. A p-value < 0.05 was considered statistically significant (p-value < 0.001 = ∗∗∗, p-value < 0.01 = ∗∗, and p-value < 0.05 = ∗). The process and study design are presented in a flowchart (**Figure 1**).

## 3 Results

### 3.1 Integration of transcriptomic datasets reveals differentially expressed genes

**Figure 2:**
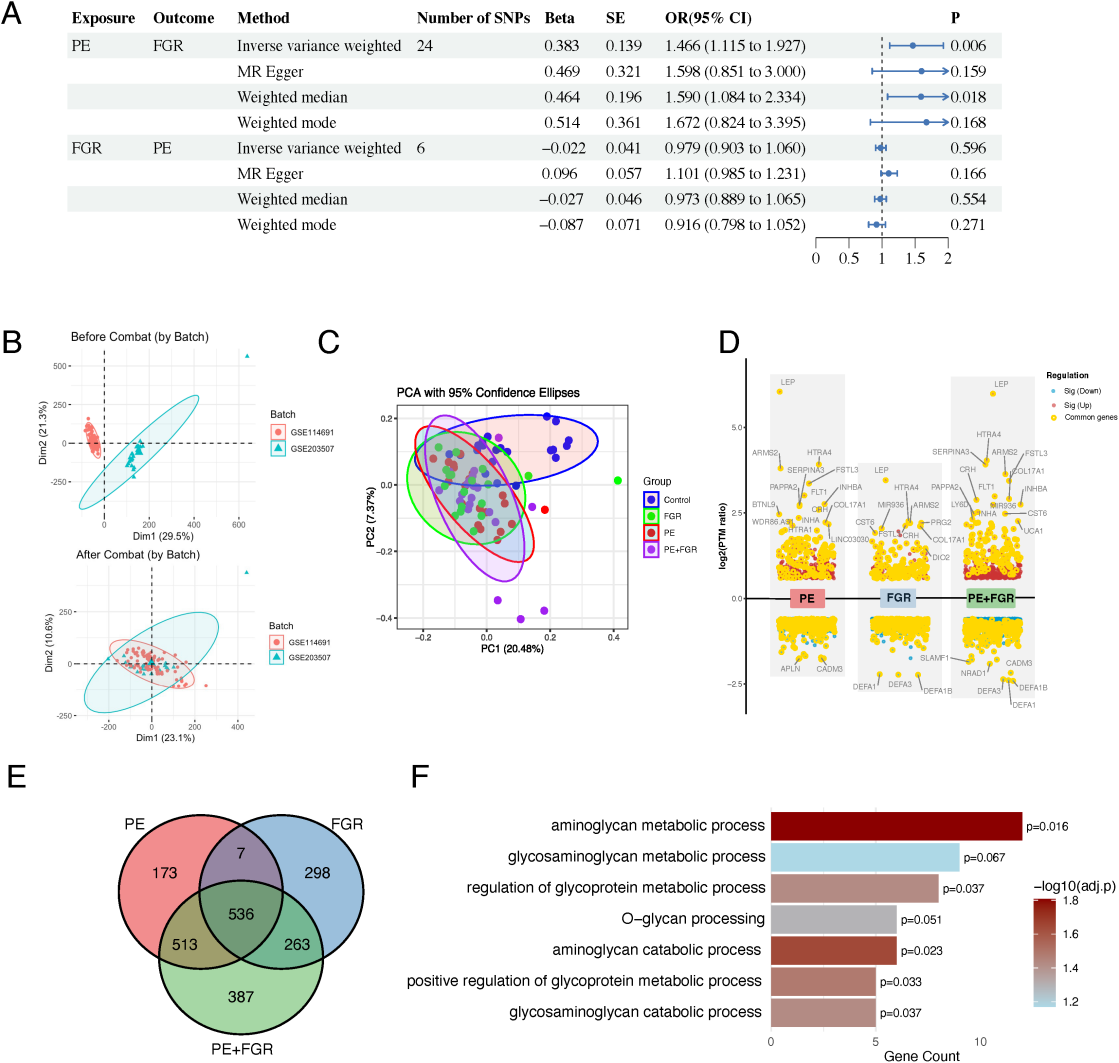
Combining different datasets and identification of DEGs. **(A)** Bidirectional Mendelian randomization (MR) analysis assessing the causal relationship between preeclampsia (PE) and fetal growth restriction (FGR). Results from multiple MR methods demonstrate a significant causal effect of PE on FGR. **(B)** Principal component analysis (PCA) plots illustrating sample distribution before (upper) and after (lower) batch effect correction, colored by dataset source. **(C)** PCA plot of integrated samples after batch correction, showing separation between disease groups (Control, FGR, PE, and PE+FGR) with 95% confidence ellipses. **(D)** Volcano plots highlighting significantly dysregulated genes in PE, FGR, and PE+FGR groups, with key glycosylation-related genes labeled. **(E)** Venn diagram of significantly dysregulated genes, identifying common and condition-specific signatures across disease groups. **(F)** Bar plot summarizing the number of dysregulated genes involved in key glycosylation-related biological processes, with color intensity representing statistical significance [-log10(adj. P)].

To investigate the potential causal relationship between PE and FGR, we first performed a bidirectional two-sample MR analysis using summary statistics from large-scale genome-wide association studies (GWAS). The IVW method, serving as the primary MR analysis, revealed a significant positive causal effect of PE on the risk of developing FGR (OR = 1.466, 95% CI: 1.115-1.927, P = 0.006). Complementary methods, including the weighted median estimator, yielded consistent directional evidence. Conversely, no significant causal association was observed from FGR to PE (IVW OR = 0.979, 95% CI: 0.903-1.060, P = 0.596) (**Figure 2A**), suggesting a unidirectional causal link from PE to FGR. This unidirectional causal link indicates that PE and FGR are not independent conditions but are causally connected, providing the rationale for the integrated transcriptomic analysis that follows and for the search for shared molecular drivers.

Given the established causal inference from PE to FGR, we next sought to elucidate the underlying molecular mechanisms by systematically profiling the placental transcriptome. Two independent placental RNA-seq datasets (GSE114691 and GSE203507), encompassing samples from control, PE, FGR, and PE+FGR, were integrated. Principal component analysis (PCA) before batch correction showed a clear separation driven by dataset origin. After applying the ComBat algorithm to remove batch effects, the samples clustered primarily by disease phenotype rather than study source (**Figure 2B**), confirming successful data harmonization. A subsequent PCA of the integrated dataset showed that the PE, FGR, and PE+FGR groups formed overlapping clusters that were clearly separated from the control group along the principal components (**Figure 2C**), indicating the shared transcriptomic features among diseased placentas.

Differential expression analysis for each disease group (PE, FGR, PE+FGR) versus controls identified numerous DEGs (adjusted P-value < 0.05, |log_2_FC| > 0.5) (**Figure 2D**). Venn diagram analysis revealed 536 DEGs shared across the three disease states (**Figure 2E**). To functionally interpret this shared signature, we performed a focused enrichment analysis specifically on the 536 common DEGs. The results revealed significant enrichment for pathways related to glycosylation processes, including “glycosaminoglycan metabolic process”, “O−glycan processing”, and “regulation of glycoprotein metabolic process” (**Figure 2F**).

### 3.2 Detection of co−expression gene modules within PE, FGR, and PE+FGR

**Figure 3:**
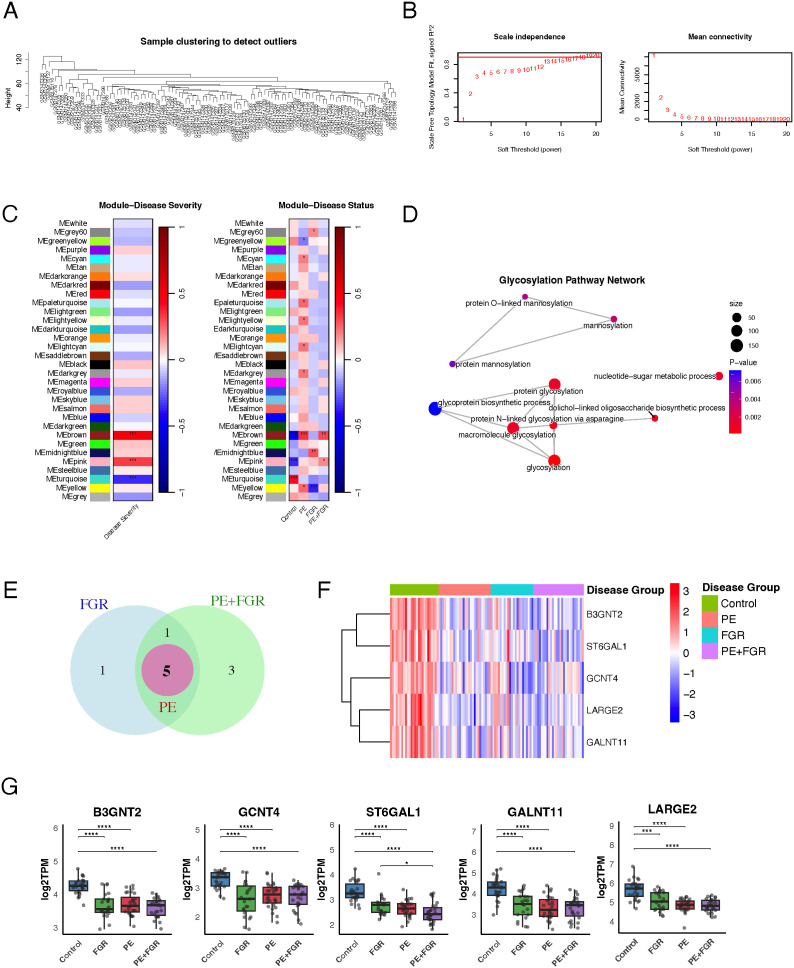
Weighted gene co-expression network analysis identifies and validates key glycosylation-related modules associated with PE and FGR. **(A)** Sample dendrogram for outlier detection. **(B)** Topology analysis for soft-thresholding power selection. **(C)** Module-trait relationship heatmap. Rows represent co-expression modules; columns represent disease severity and status. Color intensity indicates correlation. **(D)** Network of enriched glycosylation-related pathways. Node size corresponds to the number of involved genes; color indicates statistical significance [-log_10_(P-value)]. **(E)** Venn diagram showing overlaps of differentially expressed genes (DEGs) across disease groups. **(F)** Expression heatmap of key hub genes across disease groups. **(G)** Expression levels of five validated genes (B3GNT2, ST6GAL1, GALNT11, GCNT4, LARGE2) in four sample groups. Data are presented as box plots; *p < 0.05, **p < 0.01, ***p < 0.001.

Prior to co-expression network construction, the quality of the merged expression matrix was assessed, revealing no apparent outliers or missing values (**Figure 3A**). A soft-thresholding power of β = 18 was selected as the minimal value that achieved approximate scale-free topology (R² > 0.85), ensuring a biologically meaningful network (**Figure 3B**). Based on this threshold, a topological overlap matrix (TOM) was constructed, and hierarchical clustering identified 32 distinct gene modules. The module eigengenes were subsequently correlated with key clinical traits, including the disease severity score (Control=0, PE or FGR = 1, PE+FGR=2) and discrete disease states. Notably, the MEturquoise module demonstrated a significant negative correlation with the disease severity score (Cor = -0.35, P = 2.73e-4) (**Figure 3C**). Consistently, its eigengene expression was significantly higher in controls than in all disease groups (PE, FGR, and PE+FGR), suggesting a potential protective role (**Figure 3C**). To biologically characterize the MEturquoise module, enrichment analysis was performed on its 321 constituent genes. The module was significantly enriched for pathways related to protein glycosylation, N-glycan biosynthesis, and glycosaminoglycan metabolism (**Figure 3D**), corroborating the glycosylation-related signals identified in the differential expression analysis.

To refine this module toward glycosylation-focused diagnostic candidates, the 321 module genes were intersected with a curated set of 185 glycosylation-related genes (GRGs) from MSigDB, yielding 97 overlapping genes representing the glycosylation-active core of the module. Differential expression analysis of these 97 candidate GRGs showed that 7, 5, and 9 genes were significantly downregulated in the FGR, PE, and PE+FGR groups, respectively, compared with controls (**Figure 3E**). Intersection of these three disease-specific lists pinpointed five GRGs: B3GNT2, ST6GAL1, GCNT4, LARGE2, and GALNT11 (**Figure 3E**). The expression levels of all five core GRGs were markedly lower in the PE, FGR, and PE+FGR groups compared to the control group (all p < 0.001) (**Figures 3F, G**).

### 3.3 Screening for key genes based on machine learning models

**Figure 4:**
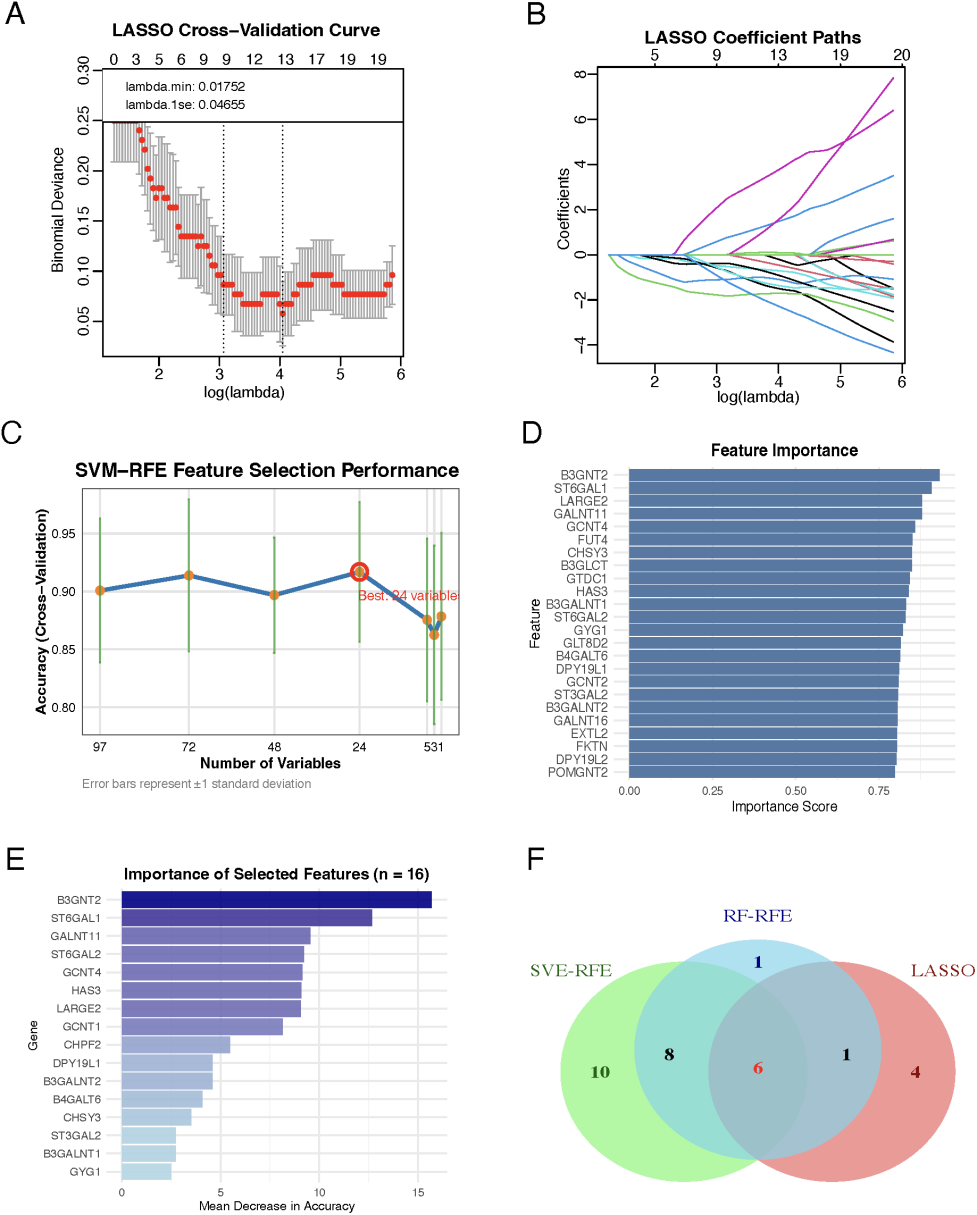
Machine learning-based identification of candidate diagnostic biomarkers. **(A)** Cross-validation curve of LASSO logistic regression. Binomial deviance is plotted against log(λ). Dashed vertical lines indicate the optimal λ values (λ.min and λ.1se) yielding minimal deviance. **(B)** LASSO coefficient paths. Gene coefficients are plotted as functions of log(λ), with the final 11 retained genes labeled. **(C)** SVM-RFE performance during 10-fold cross-validation. Cross-validation accuracy is plotted against the number of retained features. **(D)** Bar plot of feature importance scores for the 24 genes selected by SVM-RFE. **(E)** Ranked feature importance of 16 genes selected by RF-RFE. **(F)** Venn diagram summarizing the overlap of gene signatures identified by LASSO, SVM-RFE, and RF-RFE, with six candidate genes (B3GNT2, ST6GAL1, GALNT11, ST6GAL2, GCNT4, LARGE2) shared by all three methods.

To refine the diagnostic potential of the 97 intersecting GRGs, feature selection was performed using three complementary machine learning algorithms. LASSO regression identified 11 predictive genes from statistically significant univariate variables (**Figures 4A, B**). SVM-RFE with 10-fold cross-validation selected 24 diagnostic markers (**Figures 4C, D**), while RF-RFE ranked 16 DEGs by relative importance (**Figure 4E**). Integration of the three methods revealed six overlapping key genes: B3GNT2, ST6GAL1, GALNT11, ST6GAL2, GCNT4, and LARGE2 (**Figure 4F**). Further intersection of these six genes with the five previously identified shared significant DEGs yielded a final set of five core genes (B3GNT2, ST6GAL1, GALNT11, GCNT4 and LARGE2), which were used for subsequent predictive modeling.

### 3.4 Construction of diagnostic model and developing a nomogram

**Figure 5:**
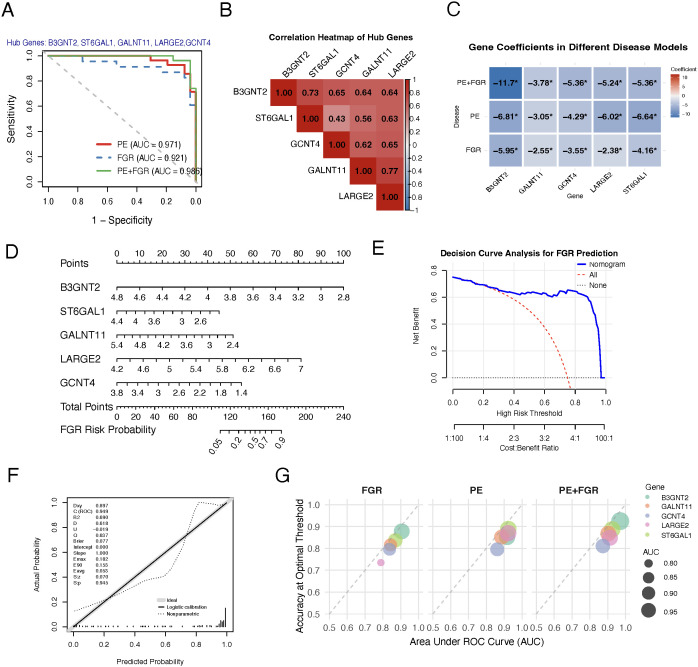
Construction and validation of a clinical prediction model based on key glycosylation-related genes. **(A)** Receiver operating characteristic (ROC) curves of the diagnostic model for distinguishing PE, FGR, and PE+FGR from controls. **(B)** Correlation between 5 diagnostic genes. **(C)** Gene coefficient matrix illustrating the contribution of each gene in the prediction models for PE, FGR, and PE+FGR. *P< 0.05 **(D)** Nomogram for individualized prediction of FGR risk based on expression levels of the five hub genes. **(E)** Decision curve analysis (DCA) for the FGR prediction model, evaluating net clinical benefit across threshold probabilities. **(F)** Calibration curve of the FGR prediction model, comparing predicted probability with observed outcome frequency. **(G)** Model performance summary, showing the relationship between accuracy and area under the curve (AUC) for each disease subtype, with point size proportional to AUC value.

ROC curve analysis demonstrated excellent performance in distinguishing disease samples (PE, FGR, and PE+FGR) from controls (**Figure 5A**). Among the five genes, expression levels showed moderate correlation, with particularly strong associations observed between B3GNT2 and ST6GAL1, and between LARGE2 and GALNT11 (**Figure 5B**). Further analysis revealed significant correlations between these hub genes and distinct placenta-related disease groups, with correlation coefficients and p-values detailed in **Figure 5C**.

Using the expression levels of these five key genes, multivariate logistic regression models were constructed. A nomogram was developed to visually predict the probability of FGR for individual samples (**Figure 5D**); nomograms for PE and PE+FGR prediction, along with their corresponding DCA curves and calibration plots, are provided in **Supplementary Figures 1A–F**. Decision curve analysis (DCA) indicated that the nomogram model provided superior net clinical benefit across a wide range of threshold probabilities (**Figure 5E**; **Supplementary Figures 1C, D**). Calibration plots showed good agreement between predicted and observed outcomes (**Figure 5F**; **Supplementary Figures 1E, F**). The five-gene combined diagnostic model exhibited excellent performance: for PE, AUC = 0.971, sensitivity = 92.9%, specificity = 92.3%, accuracy = 94.4%; for FGR, AUC = 0.921, sensitivity = 87.0%, specificity = 88.5%, accuracy = 87.8%; for PE+FGR, AUC = 0.986, sensitivity = 96.3%, specificity = 96.2%, accuracy = 98.1% (**Figure 5G**). Detailed performance metrics for individual genes across all three disease groups are provided in **Supplementary Table 3**. B3GNT2 and ST6GAL1 consistently demonstrated the highest AUC values across all disease groups, further supporting their prioritization for protein-level validation.

### 3.5 Immune infiltration analysis and the correlations between the infiltrating immune cell types in placenta

**Figure 6:**
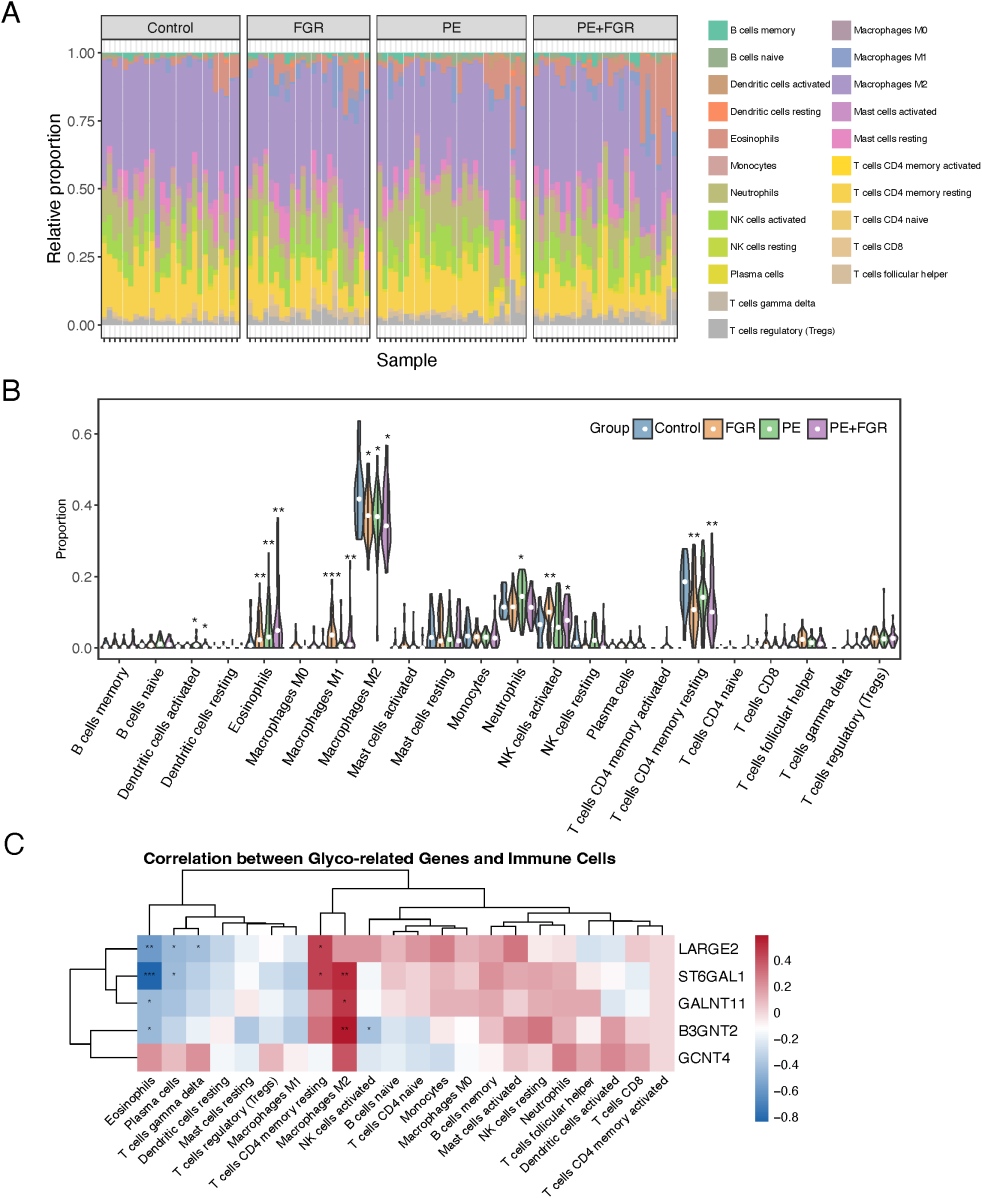
Immune cell infiltration analysis of placental tissues and correlation with glycosylation-related gene expression. **(A)** Stacked bar chart showing the relative proportions of 22 immune cell types in four groups: Control, FGR, PE, and PE+FGR. **(B)** Violin plots displaying the distribution of immune cell proportions across the four groups, with statistically significant differences indicated. **(C)** Correlation heatmap between glycosylation-related gene expression and immune cell infiltration levels. Red indicates positive correlation; blue indicates negative correlation. Key glycosylation-related genes and major immune cell types are labeled.

To characterize the immune landscape associated with placental disorders, the CIBERSORT algorithm was applied to estimate the relative proportions of 22 immune cell subtypes in placental transcriptomes across the four groups. The relative composition of these immune cells is displayed in **Figure 6A**, revealing a visually discernible shift in the immune infiltrate profile between the control and disease groups. Quantitative immune profiling identified significant inter-group differences in specific cell populations (**Figure 6B**). Alterations common to all disease groups included elevated eosinophils and reduced M2 macrophages. Condition-specific changes were also observed: increases in activated DCs (PE, PE+FGR), M1 macrophages (FGR, PE+FGR), neutrophils (PE), and activated NK cells (FGR, PE+FGR), alongside a decrease in resting CD4 memory T cells (FGR, PE+FGR).

To further investigate the relationship between glycosylation-related genes and the altered immune microenvironment in placenta, correlation analysis was performed between the expression levels of the core GRGs and the estimated immune cell fractions (**Figure 6C**). B3GNT2 expression positively correlated with M2 macrophage infiltration (r = 0.45, P < 0.001) and negatively correlated with activated NK cell (r = -0.38, P = 0.002) and eosinophil (r = -0.41, P < 0.001) infiltration. ST6GAL1 expression similarly showed a positive correlation with M2 macrophages (r = 0.39, P = 0.001) and resting CD4^+^ memory T cells (r = 0.35, P = 0.004), while its expression was negatively correlated with eosinophil (r = -0.42, P < 0.001) and plasma cell (r = -0.36, P = 0.003) infiltration. Furthermore, the expression of both LARGE2 and GALNT11 was consistently and negatively correlated with the infiltration of eosinophils. LARGE2 expression was additionally negatively correlated with gamma delta T cell and plasma cells infiltration.

### 3.6 Correlation analysis between placental biomarkers and infiltrating immune cell types in paired decidua

**Figure 7:**
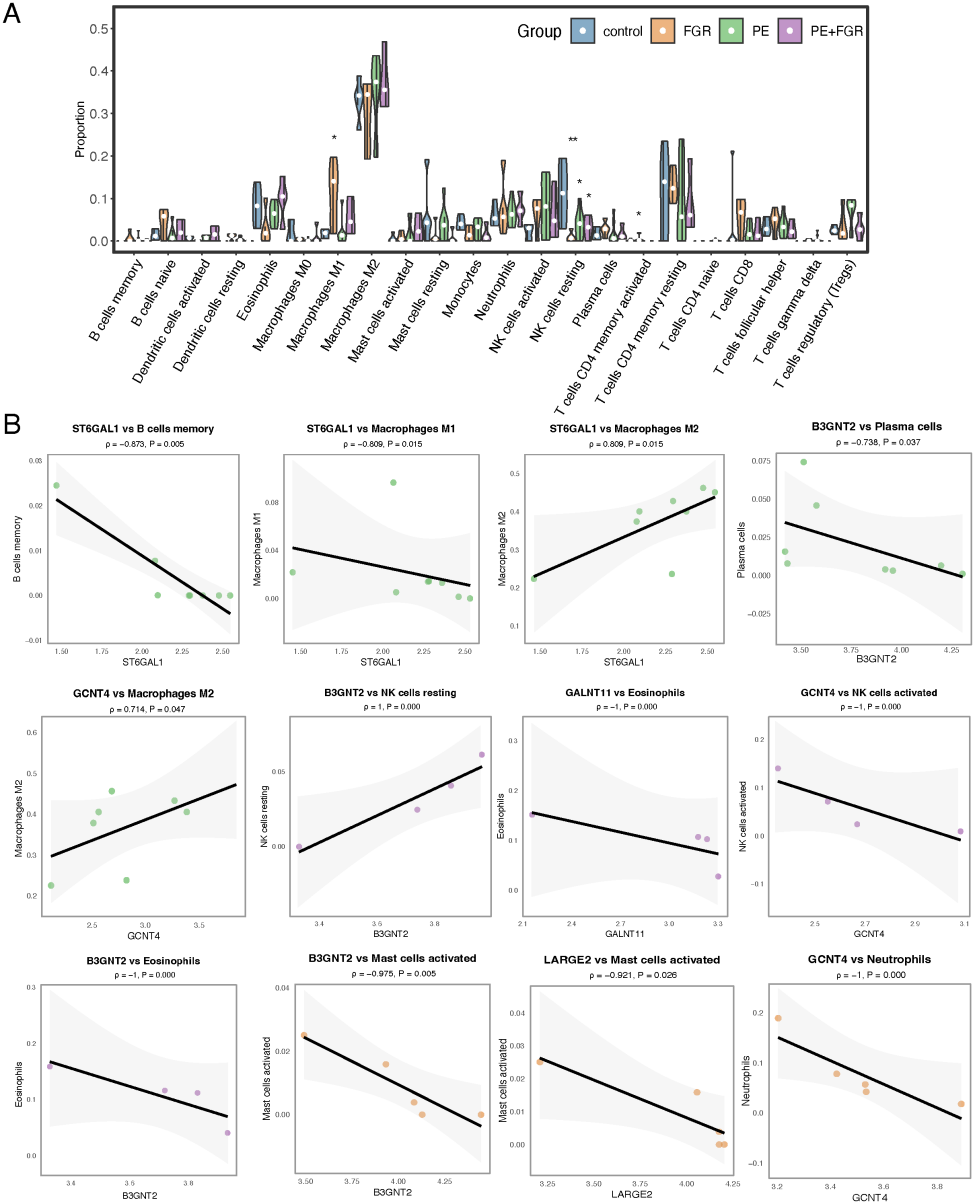
Immune cell infiltration analysis in decidual tissues and correlation with glycosylation-related gene expression. **(A)** Box plots comparing the relative proportions of 22 immune cell types across four groups: Control, FGR, PE, and PE+FGR. Statistically significant differences between groups are indicated. **(B)** Scatter plots demonstrating correlations between the expression levels of key glycosylation-related genes and the infiltration levels of their most strongly associated immune cell types. Correlation coefficients (r) and P-values are displayed for each gene-immune cell pair, highlighting both positive and negative regulatory relationships.

To investigate the association between placental dysfunction and immune dysregulation at the maternal-fetal interface, we systematically characterized the immune cell composition in decidual samples across the four clinical groups using the CIBERSORT algorithm. Comparative analysis of 22 immune cell subsets revealed significant disease-specific alterations in the decidual immune landscape (**Figure 7A**). Notably, decidua from the PE and PE+FGR groups exhibited a significant increase in the proportion of M1 macrophages (P < 0.01) and a concurrent marked decrease in resting NK cells (P < 0.01) compared to the Control group, suggesting a compromised immune-tolerant microenvironment.

Subsequently, spearman correlation analysis was performed to test the hypothesis that the expression of the five key placental glycosyltransferase DEGs modulates immune cell recruitment or polarization in the paired decidua (**Figure 7B**). Distinct, disease-specific correlation patterns were observed. In the PE group, higher placental expression of ST6GAL1 was strongly and inversely correlated with the abundance of B cell memory (ρ = -0.873, P = 0.005) and M1 macrophages (ρ = -0.809, P = 0.015) in the decidua, while it showed a significant positive correlation with M2 macrophages (ρ = 0.809, P = 0.015). Additionally, lower expression of B3GNT2 was associated with higher infiltration of plasma cells (ρ = 0.738, P = 0.037). The FGR group exhibited another unique immune-gene association profile. Higher placental expression of B3GNT2 (ρ = −0.975, P = 0.005) and LARGE2 (ρ = −0.921, P = 0.026) was inversely correlated with activated mast cell abundance in the decidua. Moreover, elevated GCNT4 expression was again associated with reduced neutrophil infiltration (ρ = −1, P < 0.001). In the more severe PE+FGR group, a distinct correlation pattern emerged. Higher placenta B3GNT2 expression correlated positively with resting NK cells (ρ = -1, P < 0.001) and negatively with eosinophils (ρ = -1, P < 0.001). Elevated expression of GALNT11 and GCNT4 was associated with reduced infiltration of neutrophils (ρ = -1, P < 0.001) and activated NK cells (ρ = -1, P < 0.001), respectively.

### 3.7 Single-cell transcriptomic profiling reveals trophoblast-specific glycosylation deficiency and intercellular communication alterations

**Figure 8:**
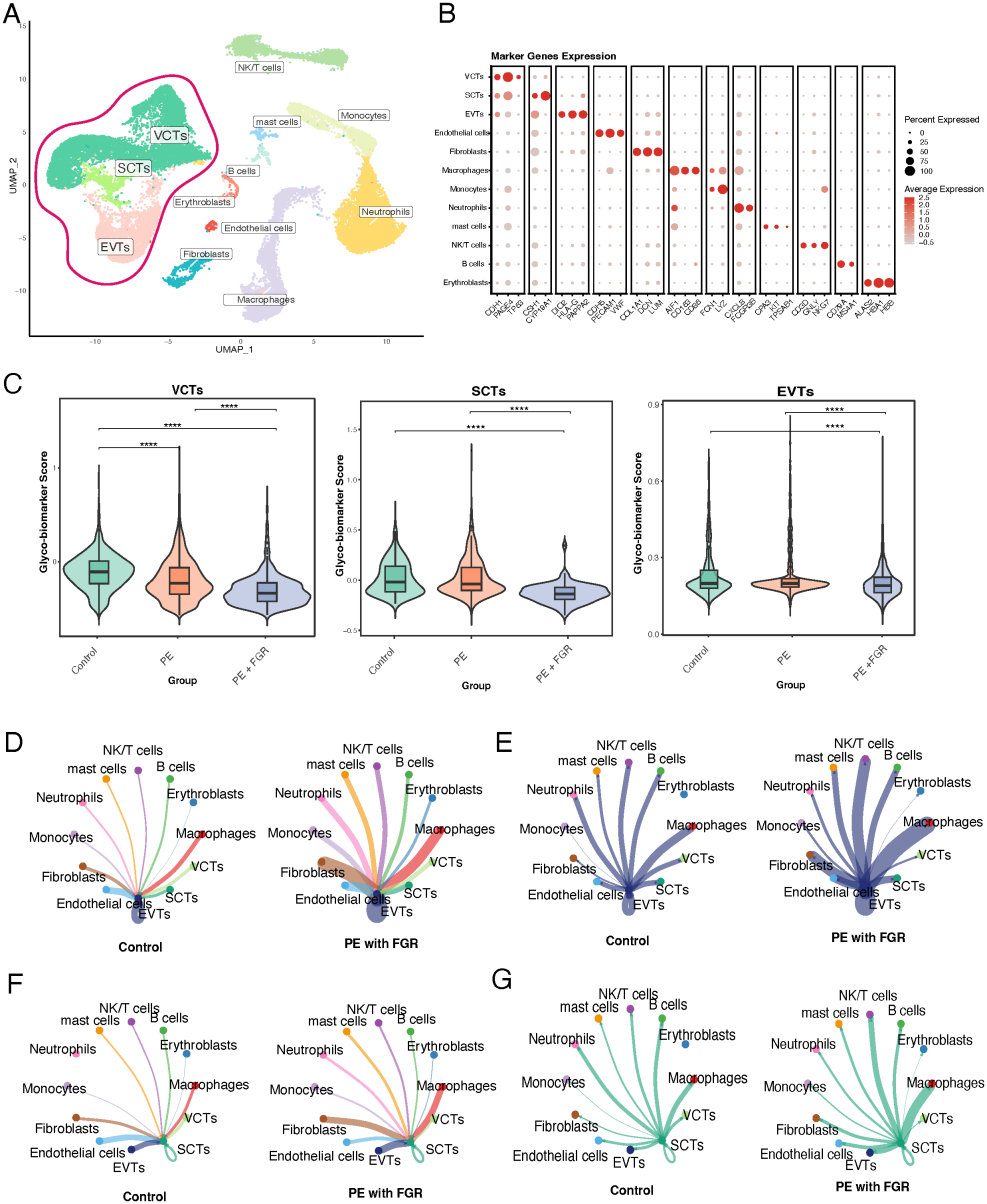
Single-cell RNA sequencing analysis validates hub gene expression in trophoblast subsets and reveals immune cell interactions. **(A)** UMAP projection of cellular landscape in placental tissue. Key trophoblast populations are annotated: VCTs (Villous cytotrophoblasts), SCTs (Syncytiotrophoblasts), and EVTs (Extravillous trophoblasts). **(B)** Dot-plot of canonical marker expression across cell clusters. **(C)** Violin plots showing the glycosylation-biomarker score in VCTs, SCTs, and EVTs across three groups: Control, PE, and PE+FGR. Statistical comparisons are indicated: ****P < 0.0001, **P < 0.01. **(D, E)** Cellular interaction networks of EVTs in Control **(D)** and PE+FGR **(E)** samples. **(F, G)** Cellular interaction networks of SCTs in Control **(F)** and PE+FGR **(G)** samples.

To dissect the cellular heterogeneity and molecular interactions underlying PE and FGR at single-cell resolution, we integrated and analyzed scRNA-seq data from PE and FGR placental samples. Unsupervised clustering and Uniform Manifold Approximation and Projection (UMAP) visualization delineated the comprehensive cellular landscape of the diseased placenta (**Figure 8A**). This included all major trophoblast lineages: villous cytotrophoblasts (VCT), syncytiotrophoblasts (SCT), and extravillous trophoblasts (EVT). The immune compartment was represented by NK/T cells, monocytes, macrophages, B cells, neutrophils, and other cell types such as erythrocytes, endothelial cells, and fibroblasts. Cluster identities were validated using canonical marker genes (**Figure 8B**). We then computed a glycosylation biomarker signature score based on five core genes (B3GNT2, ST6GAL1, GALNT11, GCNT4, LARGE2) identified previously. This score revealed a disease-specific reduction in glycosylation activity across trophoblast subpopulations (**Figure 8C**). In VCTs, the score was significantly lower in PE+FGR versus both PE (P < 0.001) and controls (P < 0.001), and lower in PE versus controls (P < 0.01). A similar pattern was observed in SCTs and EVTs, with the PE+FGR group showing the lowest scores.

To investigate whether changes in trophoblast-immune interactions align with our previous findings, we performed cell-cell communication analysis using CellChat. Comparative network analysis uncovered a profound rewiring of intercellular signaling, particularly involving immune-trophoblast crosstalk, in the PE+FGR group (**Figures 8D–G**). Specifically, we observed a significant enhancement of signaling from proinflammatory cell types toward EVTs. The incoming communication strength to EVTs from B cells, macrophages, mast cells, and neutrophils was markedly increased. Concurrently, outgoing signaling from EVTs to B cells, mast cells, and NK/T cells was also strengthened. Furthermore, the interaction network between SCTs and immune cells was altered, showing enhanced communication with NK/T cells and macrophages.

### 3.8 Real-world validation of hub gene expression

To validate the prioritized hub genes, we performed WB, IHC, and RT-qPCR on placental tissues from NP, FGR, and PE groups (clinical characteristics in **Supplementary Table 2**).

**Figure 9:**
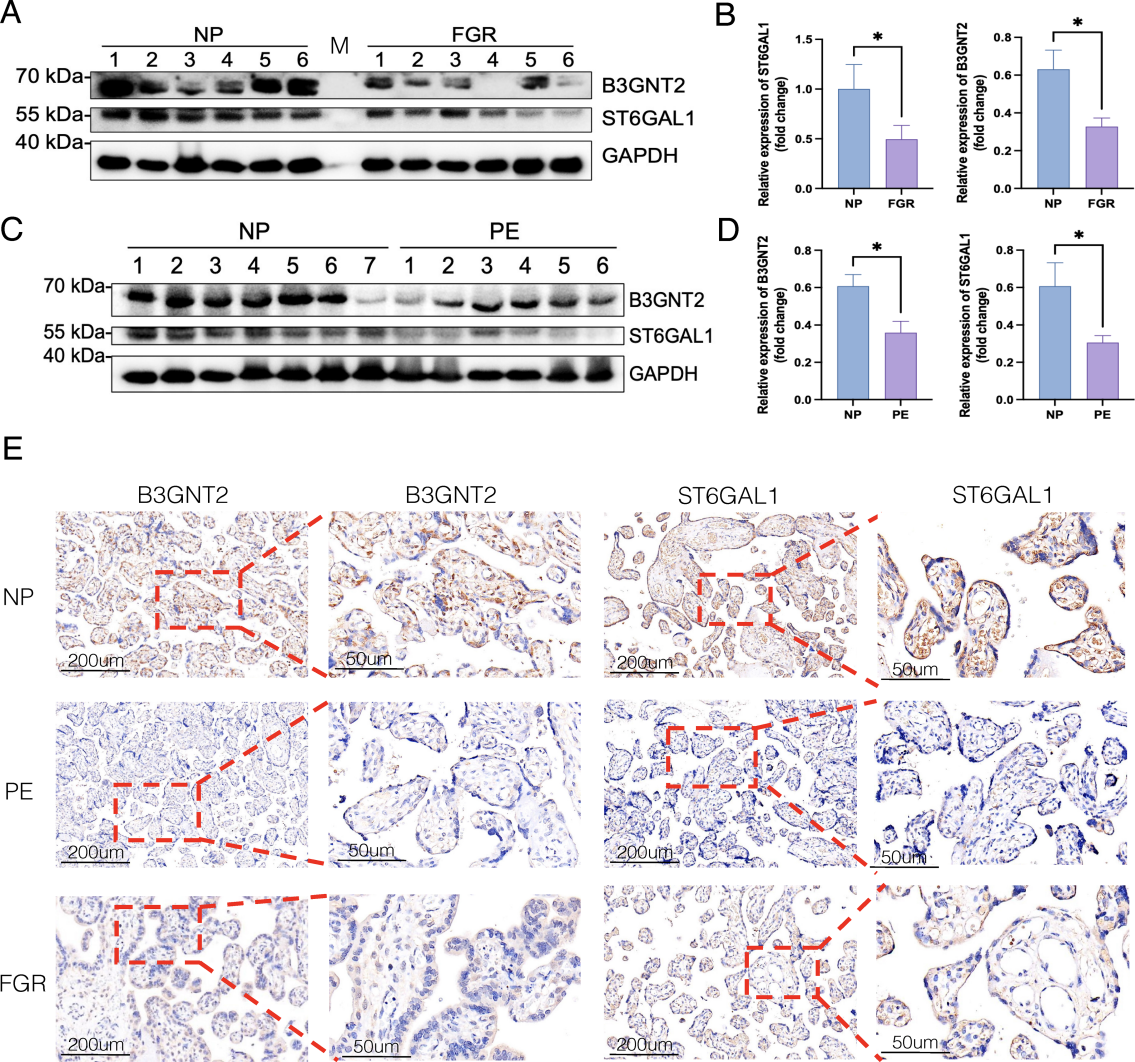
Protein-level validation of B3GNT2 and ST6GAL1 expression in placental tissues. **(A)** Representative Western blot images of B3GNT2, ST6GAL1, and GAPDH in NP (n=11), PE (n=14), and FGR (n=14) placental samples. **(B)** Quantitative analysis of B3GNT2 and ST6GAL1 protein levels in Western blot. Data are presented as mean ± SD; *p < 0.05, **p < 0.01, ***p < 0.001 vs. NP group. **(C)** Representative Western blot images of B3GNT2, ST6GAL1, and GAPDH in NP, PE, and FGR placental samples. **(D)** Quantitative analysis of B3GNT2 and ST6GAL1 protein levels in **(C)**. Data are mean ± SD; *p < 0.05, **p < 0.01, ***p < 0.001 vs. NP group. **(E)** Immunohistochemistry staining of B3GNT2 and ST6GAL1 in NP, PE, and FGR placental tissues. Scale bar: 200 μm or 50 μm.

B3GNT2 and ST6GAL1, the top two candidates across all machine learning algorithms, were prioritized for protein-level validation. WB showed significant downregulation of both proteins in FGR (ST6GAL1: p = 0.0473; B3GNT2: p = 0.0167) and PE (ST6GAL1: p = 0.0185; B3GNT2: p = 0.0101) compared to NP controls (**Figures 9A–D**). IHC localized both proteins to the trophoblast compartment (SCT and EVT), with weaker staining in PE and FGR tissues (**Figure 9E**), corroborating the WB findings.

To provide preliminary mRNA-level evidence for the remaining computationally identified candidates (GALNT11, GCNT4, LARGE2), RT-qPCR was performed for all five genes (NP n=12, PE n=14, FGR n=9; **Supplementary Figure 4**). Consistent with protein findings, B3GNT2 was downregulated in FGR (p < 0.001) and ST6GAL1 in PE (p < 0.05). Among the three additional candidates, GCNT4 and LARGE2 were significantly reduced in PE (p < 0.05), whereas GALNT11 showed a non-significant downward trend. This partial mRNA-protein discordance likely reflects post-transcriptional regulation, indicating that B3GNT2 and ST6GAL1 are the most robustly validated biomarkers while the remaining three still require further study.

## 4 Discussion

*PE and F*GR are severe pregnancy complications associated with substantial maternal and fetal morbidity (2). Both conditions share a common pathological foundation rooted in placental dysfunction, encompassing inadequate trophoblast invasion, impaired spiral artery remodeling, and the resultant placental ischemia, hypoxia, and oxidative stress (1). The causal effect of PE on FGR established by our MR analysis supports the existence of common upstream regulators shared by the two conditions; the glycosylation-related gene signature identified here represents one such candidate set of shared drivers. Currently, the early identification of PE complicated by FGR remains challenging in clinical practice, due to a lack of highly efficient and specific biomarkers and the limited accuracy of traditional screening methods (4, 13). Therefore, investigating the upstream molecular regulatory networks that drive the onset and progression of these diseases is crucial for achieving early warning and intervention.

In this study, we identified DEGs by comparing the gene expression profiles of placental tissues from the FGR, PE, and PE+FGR groups against the control group. Subsequently, we employed three machine learning algorithms to screen for key genes and construct a prognostic model. Both supervised and unsupervised machine learning techniques are capable of handling complex non-linear relationships, efficiently processing large-scale data, automatically identifying patterns, rapidly iterating and optimizing models, and providing assessments of predictive performance and accuracy. The nomogram tool offers greater quantifiability and intuitiveness, facilitating its use by clinicians. Following this, we focused the subsequent investigation on the screened key indicators, including B3GNT2, ST6GAL1, GALNT11, GCNT4 and LARGE2, to further explore the relationship between the expression of these genes and immune cell infiltration in the PE, FGR, and PE+FGR groups. The identified potential biomarkers showed significant correlations with the infiltration levels of various immune cell types in the placenta, including eosinophils, plasma cells, M1 macrophages, activated NK cells, M2 macrophages, and resting CD4^+^ memory T cells. Notably, this study represents the first protein-level validation of these glycosyltransferases in PE and FGR placental tissues.

These key prognosis genes play pivotal roles in glycan biosynthesis. β1,3-N-acetylglucosaminyltransferases (B3GNTs) are Golgi-resident glycosyltransferases involved in the biosynthesis of poly-N-acetyllactosamine chains, with B3GNT2 serving as the primary synthase (14). Deficiency of B3GNT2 markedly reduces cell surface poly-N-acetyllactosamine levels and leads to immune cell hypersensitivity and hyperreactivity. Recently, B3GNT2 has been identified as a driver of resistance to T cell-mediated cytotoxicity in human melanoma cells (15). Moreover, inhibiting the maturation of B3GNT2-mediated N-glycans can restore receptor binding and sensitivity to NK cells. Therefore, we hypothesize that at the maternal-fetal interface, trophoblasts require appropriate B3GNT2 activity to “modify” their own surface glycans, thereby moderately “calming” maternal NK cells and preventing their overactivation, which could otherwise compromise placental function.

ST6GAL1 (β-galactoside α2,6-sialyltransferase 1) catalyzes the addition of α2,6-linked sialic acids and has been increasingly linked to the regulation of immune cells (16). Several studies have shown that ST6GAL1 can influence T cells, B cells, NK cells, and monocyte-to-macrophage differentiation. Upregulation of ST6GAL1 inhibits T cell proliferation and activation, including the expression of important antigen-presenting and co-stimulatory surface receptors. Although Wang et al. reported no significant differences in ST6GAL1 mRNA expression or enzymatic activity between placental tissues from two groups of pregnant women (17), they admitted that trophoblast invasion and uteroplacental circulation are more active during early to mid-pregnancy, when ST6GAL1 activity might be particularly important. In contrast, our analysis of two large transcriptomic datasets revealed a pronounced decrease in ST6GAL1 transcription in both pure PE and PE+FGR cases. In addition, we also observed a significant reduction of this protein in PE and FGR samples compared to normal placenta, highlighting the need for further investigation.

LARGE2 is highly conserved and contains two catalytic domains with xylosyltransferase (Xyl-T) and glucuronyltransferase (GlcA-T) activities, responsible for modifying proteoglycans (PGs) with laminin-binding glycans. This bifunctional glycosyltransferase plays essential roles in basement membrane assembly and cell-matrix signaling (18). We found that this molecule was significantly downregulated in PE, FGR, and PE combined with FGR, which may lead to structural abnormalities in the placental basement membrane, impaired trophoblast invasion, and consequently contribute to defective spiral artery remodeling and insufficient placental perfusion.

O-GalNAc glycosylation is initiated by GALNT family enzymes in the Golgi apparatus. For instance, GALNT3 glycosylates adhesion proteins like E-cadherin and supports the epithelial phenotype in trophoblast lineages (19). While the role of GALNT11 in pregnancy disorders is not yet defined, studies in the kidney show that its loss disrupts megalin-mediated protein reabsorption (20), suggesting that GALNT enzymes may similarly regulate substance transport in trophoblasts.

GCNT4 is a member of the β-1,6-N-acetylglucosaminyltransferase (GCNT) family and primarily regulates protein glycosylation; it has been reported to be closely associated with gastric cancer (21). GCNT family possesses important acetylglucosaminyltransferase activity, closely related to intracellular glucosamine (O-GlcNAc) modification, further contribute to various cellular functions, including signal transduction, protein localization and stability, transcription, and cell survival.

In PE and FGR, a dysregulated inflammatory response at the maternal-fetal interface is a critical shared pathological feature (22). Our immune profiling revealed a shift towards a pro-inflammatory microenvironment in diseased placentas and decidua, characterized by an increase in M1 macrophages, activated NK cells, and neutrophils, alongside a reduction in immunoregulatory M2 macrophages, resting NK cells and resting CD4^+^ T cells. This imbalance underscores the pivotal role of macrophage polarization in the pathogenesis of PE and FGR (23, 24).

Currently, many previous studies have analyzed immune cell and gene expression correlations within either the placenta or decidua in isolation, often overlooking the dynamic crosstalk at the maternal-fetal interface (9, 25). Our findings indicate that the expression of glycosyltransferases in trophoblasts is associated with maternal immune cell infiltration, both placenta and decidua. Notably, ST6GAL1 and B3GNT2 expression was negatively correlated with the infiltration of proinflammatory cells like eosinophils and plasma cells and positively correlated with regulatory cells like M2 macrophage in the placenta. On the other hand, we found that lower placental expression of ST6GAL1 and B3GNT2 was associated with increased infiltration of memory B cells, mast cells, and eosinophils in the corresponding decidua of PE cases. These cell types are implicated in complement cascade activation and B−cell−driven inflammation, which aligns with previous reports of B−cell activation and expansion in women with PE (26, 27). This association suggests that altered trophoblast glycosylation may actively shape the immune landscape on both sides of the maternal−fetal interface.

Some potential mechanisms involve glycan-mediated immune recognition. At the maternal-fetal interface, glycans on trophoblasts can be recognized by lectin-like receptors on immune cells (5). For example, sialic acid residues, whose biosynthesis is regulated by enzymes like ST6GAL1, can engage Siglec receptors on immune cells, typically transducing inhibitory signals that maintain immune tolerance. It has been reported that highly sialylated N-glycan structures on the syncytiotrophoblast confer resistance to cytolysis mediated by NK cells and other cytolytic leukocytes (28). Additionally, aberrant glycosylation may activate the complement cascade. Supporting this, a mouse model demonstrated that sialic acid deficiency in trophoblasts triggers complement deposition such as C3 and C5, recruiting neutrophils, and ultimately leading to pregnancy failure (29). This aligns with our observed correlations between glycosyltransferase levels and the infiltration of complement-activation related cells (neutrophils, eosinophils, mast cells).

Several limitations of this study should be acknowledged. First, the lack of access to first-trimester placental tissues prevents the assessment of glycosyltransferase localization and expression patterns during early pregnancy, limiting our understanding of how these enzymes influence early placental development. Second, our study is primarily observational, focusing on associations between glycosyltransferase expression and immune cell infiltration; the causal mechanisms by which these enzymes regulate maternal-fetal immune interactions therefore remain unclear. Third, the immune deconvolution results should be interpreted as relative compositional shifts rather than absolute cell abundance, given the leukocyte-centric nature of the CIBERSORT LM22 reference matrix.

Future research should address several core questions. First, identifying the functional targets of these glycosyltransferases — specifically, which key glycoproteins on trophoblasts and immune cells are modified — will be essential. Second, delineating the downstream signaling pathways, including Siglec receptor-mediated inhibitory signals, the complement activation cascade, and intrinsic trophoblast transcription factors, will clarify the mechanistic basis of these associations (30). Third, single-cell technologies should be leveraged to distinguish the cell-specific roles of glycosylation modifications across different trophoblast subpopulations and maternal immune cell types.

In summary, this study identified five key glycosylation-related genes as potential biomarkers for PE and FGR. Among these, B3GNT2 and ST6GAL1, which demonstrated the highest diagnostic performance, were validated at both the mRNA and protein levels in independent clinical cohorts. GALNT11, GCNT4, and LARGE2 were consistently identified by three independent machine learning algorithms and confirmed at the mRNA level by qPCR in independent samples. Collectively, these findings provide new insights into the pathogenesis of placental disorders and highlight glycosylation pathways as promising candidates for early prediction and as potential targets for future therapeutic investigation.

## Data Availability

Publicly available datasets were analyzed in this study. This data can be found here: NCBI GEO repository, accession numbers GSE114691, GSE203507, GSE282038 and GSE173193.

## Supplementary material

Supplementary Figure 1 Construction of multivariate logistic regression models. A nomogram was developed to visually predict the probability of PE and PE+FGR for individual samples **(A, B)**. Decision curve analysis (DCA) indicated that the nomogram model provided superior net clinical benefit across a wide range of threshold probabilities **(C, D)**. Calibration plots showed good agreement between predicted and observed outcomes **(E, F)**.

Supplementary Figure 2 Supplementary western blot results.

Supplementary Figure 3 Complete WB blots.

Supplementary Figure 4 RT-qPCR validation of the five hub genes (B3GNT2, ST6GAL1, GALNT11, GCNT4, LARGE2) among placenta from normal pregnancy, preeclampsia and fetal growth restriction.

Supplementary Table 1 Information on microarray datasets obtained from GEO.

Supplementary Table 2 Detailed clinical characteristics of the PE, FGR and control groups.

Supplementary Table 3 Diagnostic performance metrics (AUC, sensitivity, specificity, and accuracy) of individual hub genes across PE, FGR, and PE+FGR groups.
